# A Mutation Upstream of the *rplN-rpsD* Ribosomal Operon Downregulates Bordetella pertussis Virulence Factor Production without Compromising Bacterial Survival within Human Macrophages

**DOI:** 10.1128/mSystems.00612-20

**Published:** 2020-12-08

**Authors:** Jakub Novák, David Jurnečka, Irena Linhartová, Jana Holubová, Ondřej Staněk, Daniel Štipl, Ana Dienstbier, Branislav Večerek, Nayara Azevedo, Jan Provazník, Vladimír Beneš, Peter Šebo

**Affiliations:** a Laboratory of Molecular Biology of Bacterial Pathogens, Institute of Microbiology of the Czech Academy of Sciences, Prague, Czech Republic; b Laboratory of Post-Transcriptional Control of Gene Expression, Institute of Microbiology of the Czech Academy of Sciences, Prague, Czech Republic; c Genomics Core Facility, European Molecular Biology Laboratory, Services and Technology Unit, Heidelberg, Germany; California State University, Fresno

**Keywords:** *Bordetella pertussis*, host-pathogen interactions, intracellular bacteria, macrophages, two-component regulatory systems, virulence regulation

## Abstract

The BvgS/BvgA two-component system controls expression of ∼550 genes of Bordetella pertussis, of which, ∼245 virulence-related genes are positively regulated by the BvgS-phosphorylated transcriptional regulator protein BvgA (BvgA∼P). We found that a single G-to-T nucleotide transversion in the 5′-untranslated region (5′-UTR) of the *rplN* gene enhanced transcription of the ribosomal protein operon and of the *rpoA* gene and provoked global dysregulation of B. pertussis genome expression. This comprised overproduction of the alpha subunit (RpoA) of the DNA-dependent RNA polymerase, downregulated BvgA and BvgS protein production, and impaired production and secretion of virulence factors by the mutant. Nonetheless, the mutant survived like the parental bacteria for >2 weeks inside infected primary human macrophages and persisted within infected mouse lungs for a longer period than wild-type B. pertussis. These observations suggest that downregulation of virulence factor production by bacteria internalized into host cells may enable persistence of the whooping cough agent in the airways.

**IMPORTANCE** We show that a spontaneous mutation that upregulates transcription of an operon encoding ribosomal proteins and causes overproduction of the downstream-encoded α subunit (RpoA) of RNA polymerase causes global effects on gene expression levels and proteome composition of Bordetella pertussis. Nevertheless, the resulting important downregulation of the BvgAS-controlled expression of virulence factors of the whooping cough agent did not compromise its capacity to persist for prolonged periods inside primary human macrophage cells, and it even enhanced its capacity to persist in infected mouse lungs. These observations suggest that the modulation of BvgAS-controlled expression of virulence factors may occur also during natural infections of human airways by Bordetella pertussis and may possibly account for long-term persistence of the pathogen within infected cells of the airways.

## Abstract (precis)

We show that a spontaneous mutation that upregulates transcription of an operon encoding ribosomal proteins and causes overproduction of the downstream-encoded α subunit (RpoA) of RNA polymerase causes global effects on gene expression levels and proteome composition of Bordetella pertussis. Nevertheless, the resulting important downregulation of the BvgAS-controlled expression of virulence factors of the whooping cough agent did not compromise its capacity to persist for prolonged periods inside primary human macrophage cells, and it even enhanced its capacity to persist in infected mouse lungs.

## INTRODUCTION

The Gram-negative coccobacillus Bordetella pertussis is an exclusively human pathogen and the major agent of the respiratory infectious disease called pertussis, or whooping cough (1). Pertussis used to be the primary cause of infant mortality in developed countries prior to the introduction of whole-cell pertussis vaccines (wP) in the 1950s (2–5). Despite worldwide vaccination coverage, pertussis still remains the least-controlled vaccine-preventable infectious disease, accounting for more than 12 million whooping cough cases annually worldwide and causing >100,000 pertussis deaths a year (2). Moreover, pertussis recently resurged in the most-developed countries due to the introduction of less-reactogenic acellular pertussis vaccines (aP) that confer shorter-lasting protection and fail to control transmission of the infection in vaccinated populations (6). Furthermore, the generalized pressure of the aP vaccine has promoted emergence of variants of antigens contained in the aP vaccines and has selected for globally spreading pertactin-deficient B. pertussis isolates that escape opsonization by the aP vaccine-induced antibodies (7–10).

In the course of adaptation to the human host, the recently evolved B. pertussis species lost numerous genes of its Bordetella bronchiseptica*-*like ancestor, and it represents a genetically monomorphic species that exhibits a low diversity of coding sequences and a low degree of antigenic variability between clinical isolates (6, 11). Nonetheless, the genome of B. pertussis harbors numerous copies (up to ∼240 copies) of the IS*481* insertion element, and recombination events between them generate a highly variable chromosomal architecture of the clinical isolates (12, 13). It remains to be determined whether this fluidity of genome organization and occasional duplication of portions of the genome enhance bacterial fitness and enable the bacterium to dynamically adapt to the selective pressure of the environment within host airways (11, 13–16).

B. pertussis expresses a whole array of virulence factors, producing four known immunomodulatory protein toxins, namely, the pertussis toxin (PT), the adenylate cyclase toxin-hemolysin (CyaA, ACT, or AC-Hly), the dermonecrotic toxin (DNT) and the type III secretion system effector BteA. The bacterium also employs several types of adhesins, such as fimbriae (FIM), filamentous hemagglutinin (FHA), or pertactin (PRN), and produces at least two major complement resistance factors, the autotransporter proteins Vag8 and BrkA (1, 17–20). Intriguingly, B. pertussis mutants deficient in expression of PT or PRN are still able to infect humans and can invade epithelial cells (8, 21). The bacterium produces several additional outer membrane autotransporter proteins of unknown function, and it remains to be determined whether additional toxins and virulence factors are to be discovered among the proteins of unknown function encoded in the B. pertussis genome.

The transcription of B. pertussis virulence factor genes is controlled by the master regulator of *Bordetella* virulence (*vir*), the BvgAS two-component system. This consists of a membrane-inserted BvgS sensor kinase that in the Bvg^+^ phase phosphorylates the regulatory transcription factor BvgA (22). Binding of phosphorylated BvgA (BvgA∼P) to multiple sites in the promoter regions of the so-called “*vir*-activated genes” (*vag*) then activates transcription of ∼245 virulence-related genes and indirectly represses expression of ∼326 so-called “*vir*-repressed genes” (*vrg*), which get expressed in the Bvg^−^ phase of B. pertussis (23, 24). Variation between the Bvg^+^ and Bvg^−^ phases occurs at rather high frequencies (∼10^−6^ per generation per cell) due to chromosome replication errors that cause a frameshifting expansion or retraction of a C-rich homopolymeric tract in the *bvgS* open reading frame (25, 26).

The level of activity of the BvgS/BvgA phosphorelay offers a continuum of gene expression adjustments of the BvgAS regulon (24, 27) from the Bvg^+^ “ON” state, through the intermediate Bvg^i^ “ON/OFF” state, to the avirulent Bvg^−^ “OFF” state (28, 29). The signals sensed by the BvgS kinase inside host airways remain unknown, but the levels of phosphorylation of BvgA are downmodulated, albeit incompletely, at lower growth temperatures (e.g., 24°C) (30), or in the presence of millimolar concentrations of nicotinic acid (>2 mM) or sulfate ions (>5 mM) (27, 31). The avirulent Bvg^−^ phase was found to enable survival of the animal pathogen B. bronchiseptica in the environment in association with an amoeba host (32, 33). However, it remains unclear whether modulation of BvgAS activity occurs and plays any role also during the life cycle of B. pertussis inside human airways (34). At temperatures occurring in the nasal cavity and nasopharynx of humans (∼32 to 34°C), the bacteria might adopt an intermediary Bvg^i^ phase, reducing expression of some virulence factors and upregulating production of some proteins involved in biofilm formation, such as BipA (29, 35, 36). Moreover, an intriguing observation was made during long-term follow-up of B. pertussis-infected mice and primates, where B. pertussis persistence in the nasopharynx and accumulation of Bvg^−^ phase-locked mutants was observed over several months after infection (37, 38). Downregulation of expression of most virulence factors was also recently observed in the course of B. pertussis adaptation to an intracellular niche within human THP-1 macrophages (39).

Previous reports provided evidence that the C terminus of BvgA may interact with the C terminus of the α-subunit (RpoA) of the DNA-dependent RNA polymerase during transcriptional activation of the *vag* genes (40, 41). Intriguingly, mutations that upregulated production of RpoA were found to cause a drop in transcription of the pertussis and adenylate cyclase toxin genes by an as-yet-unresolved mechanism (42). In this study, we analyzed the transcriptome and proteome of a spontaneous mutant deficient in production of a number of known virulence factors due to upregulated transcription of the operon coding for ribosomal proteins upstream of the *rpoA* gene. We show that despite a pleiotropic defect on production of known virulence factors under laboratory conditions, the mutant can persist for several weeks within primary human macrophages cultured *in vitro* and in infected mouse lungs *in vivo*.

## RESULTS

### G-to-T transversion in the 5′-UTR of a ribosomal operon causes a pleiotropic deregulation of gene expression in B. pertussis.

**FIG 1:**
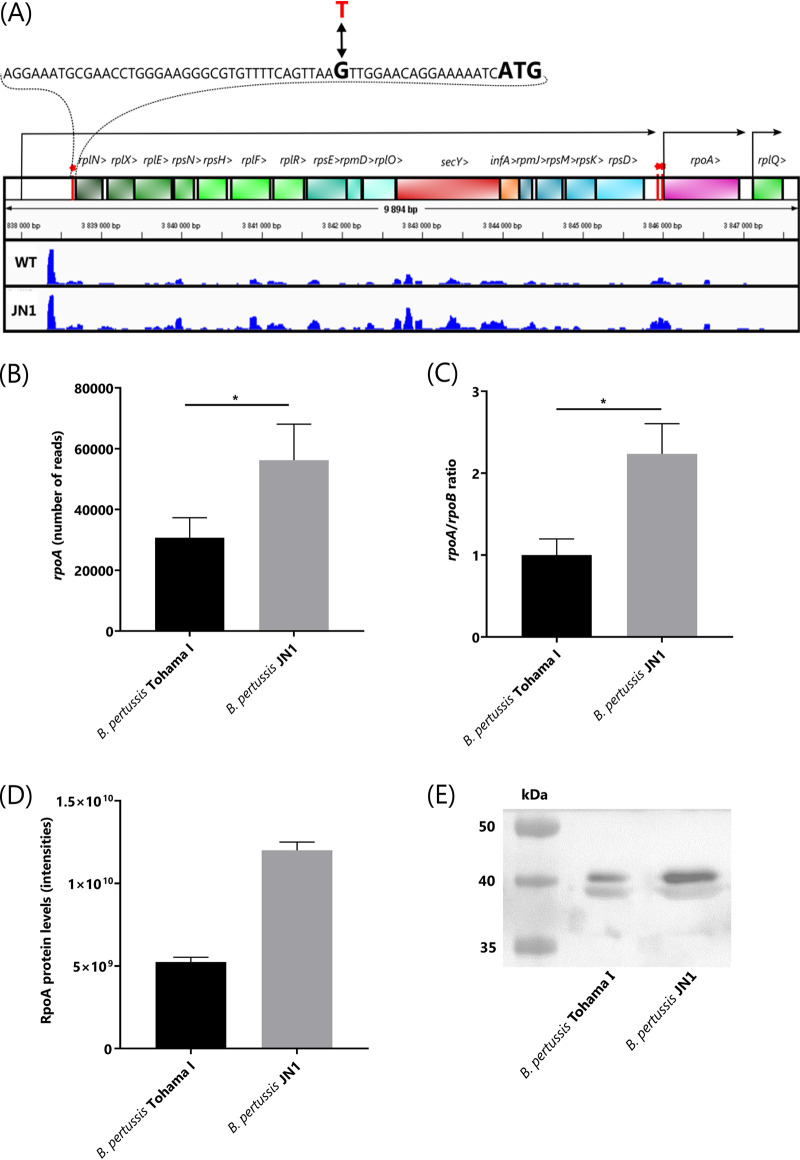
Operon structure downstream of the G-to-T transversion in the JN1 mutant strain and comparison of relative levels of *rpoA* transcript and RpoA protein in JN1 and WT B. pertussis. (A) The sequence harboring the G→T transversion SNP at position 3,838,664 of B. pertussis Tohama I chromosome in the JN1 mutant is zoomed in, indicating in bold the ATG start codon of the *rplN* gene. The Rockhopper algorithm-predicted promoter positions and transcripts are indicated by arrows. The height of the blue tracks represents the numbers of RNA-seq sequence reads that were aligned to the plus strand of the genome. The RNA-seq result for one representative pair of 4 biological replicates of B. pertussis WT and JN1 mutant transcriptomes is shown. The red bars topped by an asterisk indicate the positions at which the mutations increasing RpoA production were found by us in the JN1 mutant (a G→T transversion at position 3,838,664 upstream of *rplN*) and by Carbonetti et al. (61) in the 5′ region of the *rpoA* gene (C→T transition at 48 bp and A→G transition at 9 bp upstream of the ATG codon of *rpoA*, respectively). RNA-seq experiment result as total number of *rpoA* reads per equal amount of total bacterial RNA (B) and the *rpoA/rpoB* transcript ratios determined by qPCR analysis (C). Total production of RpoA protein in wild-type and JN1 cells determined by high-throughput proteomics analysis (D) and compared by Western blotting with an anti-RpoA polyclonal serum in equal amounts of total bacterial protein (E). *, *P* ≤ 0.05.

While deleting the *BP3063* gene from B. pertussis Tohama I chromosome, we obtained a Δ*BP3063* clone that was nonhemolytic on Bordet-Gengou agar (BGA). Intriguingly, the absence of adenylate cyclase toxin-hemolysin (ACT) production was not due to deletion of the putative deacetylase gene, since other Δ*BP3063* clones remained hemolytic. Moreover, the nonhemolytic mutant also failed to produce pertactin and pertussis toxin (not shown), whereas PCR resequencing did not reveal any mutation in the *bvgAS* locus that controls expression of *Bordetella* virulence genes (not shown). Therefore, we sequenced the genome of the nonhemolytic mutant at a high coverage (171-fold) using Illumina technology (ENA project PRJEB38438) and aligned the reads to the Tohama I reference genome sequence. This analysis revealed a single G-to-T base transversion at position 3,838,664, hence, in the 5′-untranslated region (5′-UTR) of the *BP3626*-*rplN* gene of the operon encoding ribosomal proteins (Fig. 1A). To confirm that the G-T transversion located 18 bases upstream of the start codon of *rplN* accounted for the nonhemolytic phenotype, we introduced the point mutation into the chromosome of the parental B. pertussis Tohama I strain. Indeed, the generated sequence-confirmed JN1 mutant was nonhemolytic on BGA plates (not shown) and grew slightly faster in standard Stainer-Scholte medium (SSM) (43) than the wild-type bacteria (see Fig. S1 in the supplemental material).

10.1128/mSystems.00612-20.1 FIG S1 Comparison of growth rates of B. pertussis Tohama I and JN1 mutant strains. Cultures were grown in standard Stainer-Scholte medium (SSM). The experiment was done in duplicates. Download FIG S1, TIF file, 0.3 MB. Copyright © 2020 Novák et al. 2020 Novák et al. This content is distributed under the terms of the Creative Commons Attribution 4.0 International license.

To determine the extent to which the G-T transversion in the 5′-UTR of *rplN* affected virulence factor production, we compared the proteome of the JN1 mutant to that of Tohama I bacteria grown under identical conditions. Altogether 1,466 individual proteins were identified in the proteomic data set generated on 3 replicate culture samples of the two strains (see Table S1). This covered almost 45% of the theoretical proteome of B. pertussis. Based on a *t* test, 472 of the identified proteins were differentially expressed between the wild-type and JN1 strains and another 61 proteins were detected in one proteome but not the other (presence/absence). When a secretome analysis was performed using concentrated culture supernatants (see Table S2), 804 proteins were detected in total, and 292 of them were differentially expressed between the wild type and the JN1 mutant, based on *t* test analysis. Sixty-seven other proteins were then reproducibly present/absent in one of the two secretomes. Hence, the G-T transversion at position 3,838,664 of the JN1 chromosome had a truly pleiotropic impact on expression of the genome of B. pertussis.

10.1128/mSystems.00612-20.2 TABLE S1 Total proteome data from bacterial lysates of B. pertussis Tohama I and JN1 mutant strains. Download Table S1, XLSX file, 0.5 MB. Copyright © 2020 Novák et al. 2020 Novák et al. This content is distributed under the terms of the Creative Commons Attribution 4.0 International license.

10.1128/mSystems.00612-20.3 TABLE S2 Proteomic analysis of secretome of B. pertussis Tohama I and JN1 mutant strains. Download Table S2, XLSX file, 0.3 MB. Copyright © 2020 Novák et al. 2020 Novák et al. This content is distributed under the terms of the Creative Commons Attribution 4.0 International license.

### Production of most virulence factors is strongly downregulated in the JN1 mutant due to reduced levels of the BvgA protein.

Since the G-T transversion did not occur in any known transcriptional regulator gene, we examined if its pleiotropic effect on proteome composition may have resulted from alteration of any potential regulatory small RNA (sRNA) sequence. Towards this aim, we compared the transcriptomes of the wild-type and JN1 strains by unbiased comparative transcriptome sequencing (RNA-seq) analysis (see Tables S3 and S4). To ensure that the maximum number of sRNA molecules shorter than 50 nucleotides was retained, no length restrictions on the size of analyzed RNA molecules were applied, and cDNA libraries were prepared from total RNA of five wild-type and four JN1 strain replicate samples. Upon deep sequencing, the candidate sense and antisense sRNA sequences and the operon structures were identified using the Rockhopper software algorithms (44, 45). As summarized in Table S5, the G-to-T transversion at position 3,838,664 most likely did not affect the sequence of any sRNA transcript, as the most closely located sRNA sequence was identified between bases 3,838,293 and 3,838,561 on the plus strand of the chromosome. The G-T transversion also did not affect transcription of the *BP3624* gene in the opposite direction from the ribosomal operon (Table S4).

10.1128/mSystems.00612-20.4 TABLE S3 RNA-seq analysis of B. pertussis Tohama I and JN1 mutant strains—total counts. Download Table S3, XLSX file, 0.3 MB. Copyright © 2020 Novák et al. 2020 Novák et al. This content is distributed under the terms of the Creative Commons Attribution 4.0 International license.

10.1128/mSystems.00612-20.5 TABLE S4 RNA-seq analysis of B. pertussis Tohama I and JN1 mutant strains—analysis of differential expression. Download Table S4, XLSX file, 0.4 MB. Copyright © 2020 Novák et al. 2020 Novák et al. This content is distributed under the terms of the Creative Commons Attribution 4.0 International license.

10.1128/mSystems.00612-20.6 TABLE S5 Analysis of RNA-seq data—identification of transcript boundaries and small RNAs. Download Table S5, XLSX file, 0.5 MB. Copyright © 2020 Novák et al. 2020 Novák et al. This content is distributed under the terms of the Creative Commons Attribution 4.0 International license.

However, due to the G-T transversion in the 5′-UTR of *rplN*, transcription of the downstream ribosomal operon genes was increased by a factor of two to three (Table 1). This translated only in part into an alteration of the levels of produced ribosomal proteins (Table 1). For example, the *rplN*-encoded 50S ribosomal protein L14 could not itself be classified as significantly upregulated in JN1. Some of the downstream-encoded proteins then classified as insignificantly upregulated and some as downregulated (Table 1). However, the expression of the immediately downstream-located *rpoA* gene (Fig. 1), encoding the alpha subunit of the DNA-directed RNA polymerase (RpoA), was statistically significantly upregulated 2- to 3-fold on both transcript and protein levels (Table 1), as also verified by quantitative PCR (qPCR) (Fig. 1B and C) and Western blot analysis (Fig. 1D and E), respectively.

**TABLE 1:** Proteomic and transcriptomic analyses of expression levels of the ribosomal operon genes located downstream to the G-to-T transversion at position 3,838,664 in the JN1 mutant chromosome

Gene	Protein	Expression level^*a*^					
		Transcriptome			Proteome		
		FC	JN1/WT	*P* value	FC	JN1/WT	*P* value
*rplN*	50S ribosomal protein L14	2.3	↑	8.1E−26	1.6	NS	2.11E−01
*rplX*	50S ribosomal protein L24	2.8	↑	5.3E−30	1.0	NS	9.50E−01
*rplE*	50S ribosomal protein L5	2.5	↑	4.6E−25	1.3	NS	6.43E−02
*rpsN*	30S ribosomal protein S14	3.1	↑	1.6E−21	ND		ND
*rpsH*	30S ribosomal protein S8	2.2	↑	1.1E−13	1.3	↑	3.51E−02
*rplF*	50S ribosomal protein L6	2.5	↑	1.9E−21	1.4	NS	8.43E−01
*rplR*	50S ribosomal protein L18	2.7	↑	5.4E−36	2.7	↓	5.29E−04
*rpsE*	30S ribosomal protein S5	2.1	↑	9.3E−09	2.4	↓	7.73E−03
*rpmD*	50S ribosomal protein L30	2.6	↑	1.8E−35	ND		ND
*rplO*	50S ribosomal protein L15	3.2	↑	6.3E−74	1.7	NS	8.79E−01
*secY*	Protein translocase subunit SecY	2.1	↑	8.5E−16	ND		ND
*infA2*	Translation initiation factor IF-1 2	1.8	↑	1.5E−12	2.6	NS	6.25E−01
*rpmJ*	50S ribosomal protein L36	2.1	↑	3.5E−28	ND		ND
*rpsM*	30S ribosomal protein S13	2.7	↑	2.2E−70	2.4	↓	3.56E−05
*rpsK*	30S ribosomal protein S11	2.2	↑	5.3E−71	2.4	↓	2.64E−03
*rpsD*	30S ribosomal protein S4	2.9	↑	2.7E−57	1.7	↓	1.33E−02
*rpoA*	DNA-directed RNA polymerase subunit alpha	1.9	↑	2.0E−44	2.3	↑	5.94E−02
*rplQ*	50S ribosomal protein L17	1.3	↑	2.6E−10	1.3	NS	1.01E−01

a FC, fold change difference represents the absolute ratio of the values of the tested samples. For the transcriptome, it was derived from the “log2FoldChange” value from DESeq2. For the proteome, it was derived as a fold change difference of median values of intensities. ND, not detected. JN1/WT, change trend observed for JN1 mutant compared to the WT, indicated as higher (↑) or lower (↓) mRNA/protein levels in the JN1 mutant compared to that in the wild-type strain. NS, not statistically significantly different.

Importantly, as assessed by label-free proteomics, the JN1 mutant produced >2-fold smaller relative amounts of the BvgA and BvgS proteins that govern expression of virulence-related genes and the production of the bona fide virulence factors of B. pertussis (Table 2). Indeed, the transcriptomic and proteomic examinations confirmed that the relative amounts of the adenylate cyclase toxin-hemolysin (ACT) protein were strongly reduced in the biomass and culture supernatants of the JN1 mutant, explaining its nonhemolytic phenotype (Table 2). Furthermore, a significant downregulation of production of other toxin proteins, adhesins, or of components of the type III and type IV secretion systems was observed in JN1 (Table 2). The extent of downregulation varied between the virulence-related proteins, and even if some of them accumulated at close to wild-type levels in the biomass of bacterial cells, their amounts secreted into culture supernatants of the JN1 mutant were much decreased (Table 2). This was particularly true for pertussis toxin and the strongly reduced amounts of its S1 to S4 subunits in culture supernatants. The low level of PT secretion was likely due to a strong downregulation of the PtlC, -F, and -G protein components of the type IV system involved in pertussis toxin excretion from bacterial periplasm (46). Similarly, the amount of the fimbriae protein Fim2 was strongly reduced, as were the amounts of the autotransporters and complement resistance proteins BrkA and Vag8 anchored in the bacterial outer membrane or of the type III secretion system proteins BopB, BopD, BscC, BscE, BscL, BscP, BscQ, and BscU. Intriguingly, we also observed a significant downregulation of the highly conserved 86-kDa Tex protein involved in the regulation of toxin expression in B. pertussis and Clostridium perfringens (47, 48). In contrast, proteins encoded by *BP3399* (putative zf-CHCC domain-containing protein), *BP1315* (universal stress family protein), and *BP3501* (putative *Bordetella* uptake gene) were strongly overproduced in the JN1 mutant (Table S1).

**TABLE 2:** Virulence factors significantly deregulated on protein level

Gene	Protein	Expression level^*a*^					
		Transcriptome		Proteome		Secretome	
		FC	JN1/WT	FC	JN1/WT	FC	JN1/WT
*bipA*	BipA, *Bordetella* Bvg-intermediate phase outer membrane protein involved in biofilm formation	2.4	**↑**	2.4	**↑**	2.1	**↑**
*BP0529*	Autotransporter	1.6	**↑**	4.2	**↑**	1.6	**↓**
*sphB3*	Serine protease	1.1	**↑**	ND		P/A	**↑**
*bopB*	Type III secreted protein B	10.3	**↓**	P/A	**↓**	P/A	**↓**
*bopD*	Type III secreted protein D	7.7	**↓**	12.1	**↓**	3356.8	**↓**
*bopN*	Type III secreted protein N	7.5	**↓**	ND		P/A	**↓**
*BP0499*	Uncharacterized protein	7.4	**↓**	10.1	**↓**	P/A	**↓**
*BP1251*	Putative toxin	5.4	**↓**	7.6	**↓**	ND	
*BP1252*	Putative exported protein	5.8	**↓**	4.3	**↓**	4.9	**↓**
*BP2265*	Uncharacterized protein	6.0	**↓**	P/A	**↓**	ND	
*brkA*	BrkA autotransporter involved in complement resistance	7.4	**↓**	3.1	**↓**	4.7	**↓**
*bscC*	Type III secretion system protein C	9.0	**↓**	P/A	**↓**	ND	
*bscE*	Type III secretion system protein E	4.0	**↓**	24.2	**↓**	ND	
*bscL*	Type III secretion system protein L	6.6	**↓**	P/A	**↓**	ND	
*bscP*	Type III secretion system protein P	6.9	**↓**	P/A	**↓**	ND	
*bscQ*	Type III secretion system protein Q	7.2	**↓**	P/A	**↓**	ND	
*bscU*	Type III secretion system protein U	6.7	**↓**	P/A	**↓**	ND	
*bvgA*	*Bordetella* virulence gene regulator protein BvgA	2.4	**↓**	2.5	**↓**	2.4	**↓**
*bvgR*	*Bordetella* avirulence gene repressor BvgR	1.2	**↓**	1.5	**↓**	ND	
*bvgS*	*Bordetella* virulence gene regulator kinase BvgS	2.2	**↓**	2.5	**↓**	ND	
*cyaA*	Bifunctional adenylate cyclase toxin-hemolysin	5.1	**↓**	448.9	**↓**	62.8	**↓**
*dnt*	Dermonecrotic toxin	5.9	**↓**	P/A	**↓**	2.3	**↓**
*fhaB*	Filamentous hemagglutinin	1.6	**↓**	1.9	**↓**	3.2	**↓**
*fhaC*	Filamentous hemagglutinin transporter protein FhaC	2.8	**↓**	1.9	**↓**	2.4	**↓**
*fhaE*	Protein FhaE	2.5	**↓**	1.6	**↓**	1.8	**↓**
*fhaL*	Adhesin	2.4	**↓**	P/A	**↓**	6.8	**↓**
*fhaS*	Adhesin	2.3	**↓**	P/A	**↓**	4.8	**↓**
*fim2*	Serotype 2 fimbrial subunit	5.8	**↓**	2.6	**↓**	114.1	**↓**
*fimB*	Chaperone protein FimB/FhaD	2.6	**↓**	1.4	**↓**	ND	
*prn*	Pertactin autotransporter adhesin	2.7	**↓**	ND		2.5	**↓**
*ptlC*	Type IV secretion system protein PtlC	4.1	**↓**	P/A	**↓**	P/A	**↓**
*ptlF*	Type IV secretion system protein PtlF	4.9	**↓**	2.9	**↓**	2.9	**↓**
*ptlG*	Type IV secretion system protein PtlG	4.5	**↓**	P/A	**↓**	P/A	**↓**
*ptxA*	Pertussis toxin subunit 1	4.4	**↓**	ND		62.7	**↓**
*ptxB*	Pertussis toxin subunit 2	4.0	**↓**	ND		188.7	**↓**
*ptxC*	Pertussis toxin subunit 3	3.8	**↓**	ND		163.9	**↓**
*ptxD*	Pertussis toxin subunit 4	3.8	**↓**	ND		420.5	**↓**
*sphB1*	Autotransporter subtilisin-like protease	4.7	**↓**	3.4	**↓**	3.6	**↓**
*tcfA*	Tracheal colonization factor	49.8	**↓**	14.4	**↓**	27.6	**↓**
*tex*	Transcription accessory protein Tex	1.8	**↓**	5.5	**↓**	P/A	**↓**
*vag8*	Autotransporter protein Vag8, complement resistance	93.8	**↓**	19.1	**↓**	119.6	**↓**

a FC, fold change difference represents the absolute ratio of the values of the tested samples. For the transcriptome, it was derived from the “log2FoldChange” value from DESeq2. For the proteome and secretome, it was derived as a fold change difference of median values of intensities. ND, not detected; P/A, significance determined based on presence/absence criterion, otherwise, only proteins with a *P* value of ≤0.05 are shown. JN1/WT, change trend observed for JN1 mutant compared to the WT, indicated as higher (↑) or lower (↓) mRNA/protein levels in the JN1 mutant compared to that in the wild-type strain.

Nevertheless, the downregulation of the BvgA, BvgS, and BvgR proteins in JN1 was not sufficient for a full upregulation of transcription of the >300 previously identified *vrg* genes expressed in the Bvg^−^ phase (23, 49). Transcription of only 144 *vrg* genes was significantly upregulated (fold change [FC] ≥ 1.5, adjusted *P* < 0.05) in JN1 (see Table S6), with the most upregulated *vrg* genes (FC ≥ 2.5) listed in Table 3. The level of residual transcription of some 36 *vrg* genes in JN1 remained as low as or even lower than in the wild-type strain (Table S6). For example, the *vrg-6* gene was not upregulated in JN1 (Table S6), in line with the rather modest downregulation of the BvgR phosphodiesterase protein that prevents activation of *vrg-6* transcription (34).

**TABLE 3:** Comparison of gene expression and protein levels of selected *vir*-repressed genes (*vrgs*) in wild-type and JN1 strains

Gene	Name	Product	Expression level^*a*^					
			Transcriptome		Proteome		Secretome	
			FC	JN1/WT	FC	JN1/WT	FC	JN1/WT
*BP1311*		Membrane protein	4.1	**↑**	NS	ND	NS	ND
*BP2922*	*bfrG*	TonB-dependent receptor	3.6	**↑**	NS	ND	NS	ND
*BP3501*	*vrg-24*	Hypothetical protein	3.5	**↑**	4.4	↑	4.9	↑
*BP3635*	*rplO*	50S ribosomal protein L15	3.2	**↑**	NS	ND	NS	ND
*BP2671*		Hypothetical protein	3.1	**↑**	2.8	↑	4.6	↑
*BP1991*		Flp pilus assembly membrane protein	3.0	**↑**	NS	ND	NS	ND
*BP3671*		Glycosyl transferase family protein	2.9	**↑**	NS	ND	NS	ND
*BP2923*		Lipoprotein	2.8	**↑**	NS	ND	NS	ND
*BP3497*		Hypothetical protein	2.8	**↑**	P/A	↑	NS	ND
*BP3627*	*rplX*	50S ribosomal protein L24	2.8	**↑**	NS	ND	NS	ND
*BP3440*		Putative exported protein	2.8	**↑**	2.6	↑	3.8	↑
*BP3498*		Hypothetical protein	2.8	**↑**	2.0	↑	NS	ND
*BP3500*		FAD-binding dehydrogenase	2.7	**↑**	NS	ND	NS	ND
*BP1996*		Putative type II secretion system protein	2.6	**↑**	NS	ND	NS	ND
*BP1996*		Putative type II secretion system protein	2.6	**↑**	NS	ND	NS	ND
*BP1532*		Amino acid ABC transporter substrate-binding protein	2.5	**↑**	2.8	↑	2.3	↑

a FC, fold change difference represents the absolute ratio of the values of the tested samples. For the transcriptome, it was derived from the “log2FoldChange” value from DESeq2. For the proteome and secretome, it was derived as a fold change difference of median values of intensities. NS, not significantly different; P/A, significance determined based on presence/absence criterion, otherwise, only proteins with a *P* value of ≤0.05 are shown. JN1/WT, change trend observed for JN1 mutant compared to the WT, indicated as higher (↑) or lower (↓) mRNA/protein levels in the JN1 mutant than in the wild-type strain. ND, not detected.

10.1128/mSystems.00612-20.7 TABLE S6 List of *vrg* genes identified by Moon et al. (23) and/or de Gouw et al. (54) which were significantly deregulated in JN1. Download Table S6, XLSX file, 0.03 MB. Copyright © 2020 Novák et al. 2020 Novák et al. This content is distributed under the terms of the Creative Commons Attribution 4.0 International license.

### JN1 produces small amounts of BvgA∼P.

**FIG 2:**
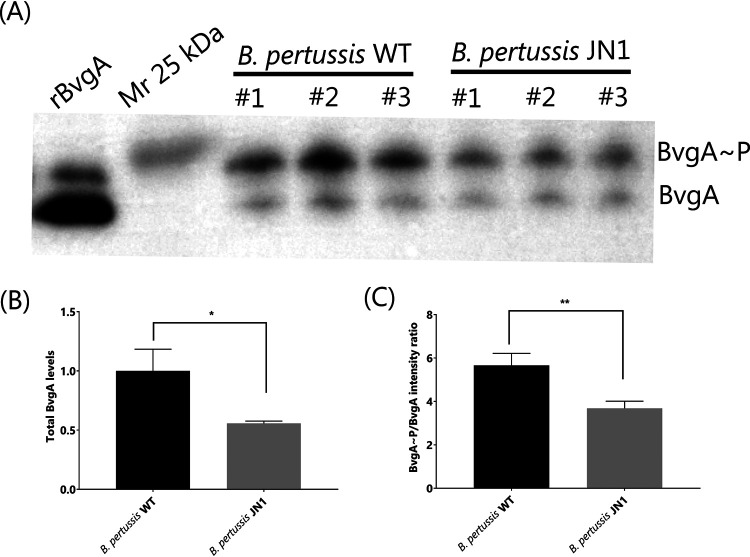
BvgA production and phosphorylation in the JN1 mutant. (A) Production and phosphorylation of BvgA. Equal amounts of total bacterial protein from three independent cultures of wild-type and JN1 strains were separated using a 12.5% SuperSep Phos-tag gel, and the BvgA and BvgA∼P proteins were detected by Western blotting using a polyclonal anti-BvgA antibody with chemiluminescence detection; 0.1 μg of purified recombinant 6×His-BvgA protein (rBvgA) was used as a positive control. (B) Total detected amounts of BvgA in the JN1 cells normalized to the total BvgA amount. (C) Ratios of phosphorylated BvgA∼P versus nonphosphorylated BvgA amounts, as determined by quantitative luminometric analysis of Western blot signals. *, *P* ≤ 0.05; **, *P* ≤ 0.01.

It was important to examine whether the generalized decrease of virulence factor production in the JN1 mutant was due to reduced levels of the phosphorylated BvgA (BvgA∼P) transcriptional regulator that is crucial for activation of virulence gene transcription. Therefore, we compared the relative amounts of BvgA∼P protein in the JN1 mutant and in wild-type bacteria using total protein extracts separated on 12.5% SuperSep Phos-tag gels that allow BvgA∼P to be resolved from BvgA (50). The two forms of the protein could thus be immunodetected by Western blots with a BvgA-specific antibody (51). As shown in Fig. 2A and B, an approximately 2-fold lower chemiluminescent signal of total BvgA protein was detected for equal amounts of total biomass protein of JN1 cells than for the wild-type B. pertussis cells, in agreement with the results of the label-free proteomic analysis (cf. Table 2). Moreover, the ratio of the detected BvgA∼P form to the nonphosphorylated BvgA protein was reduced by approximately 30% in the JN1 mutant (Fig. 2C), likely due to a reduced level of the BvgS kinase in JN1 cells (cf. Table 2). Hence, the total cytosolic concentration of BvgA∼P can be estimated to have been approximately 3-fold lower in the JN1 cells than in wild-type bacteria cultured under identical conditions.

To test the hypothesis that the overproduced free RpoA deregulated virulence gene expression in JN1 by sequestering BvgA and/or other transcription factors into nonproductive complexes, we immunoprecipitated RpoA from lysates of wild-type or JN1 cells with an anti-RpoA polyclonal serum (52). The spectra of proteins pulled down by RpoA were compared, and due to the extreme sensitivity of mass spectrometric detection, a total of 530 proteins were identified in at least two replicates of the precipitates from either wild-type or JN1 but not in the negative control (see Table S7). Only proteins detected in at least two replicates of a sample set were considered further, and the significance of differences in protein amounts in the immunoprecipitates was assessed by a *t* test and by the presence/absence criterion. This identified 36 RpoA-associated proteins that were significantly enriched in the JN1 samples and 35 proteins that were present in significantly reduced amounts compared to the composition of the precipitates from wild-type bacteria (Table S7). Among the proteins enriched in the JN1 samples were several proteins involved in transcriptional regulation, such as the BP2216 protein from the MarR family of transcriptional regulators, a universal stress family protein BP1315, and the ferric uptake transcriptional regulator protein (Table 4). Importantly, the RpoB subunit of the RNA polymerase was not detected in the precipitates from JN1 lysates, indicating that the anti-RpoA antibody pulled down preferentially the free overproduced RpoA protein and not the RNA polymerase complexes. Increased relative amounts of RpoA were pulled down from JN1 lysates compared to that from wild-type (WT) lysates (Table S7), whereas reduced amounts of the BvgA protein were detected in the JN1 precipitates compared to that in the wild-type sample, even if the decrease of the BvgA amount was not statistically significant (Table 4). The result, thus, did not enable us to conclude whether the overproduced free RpoA formed complexes with BvgA.

**TABLE 4:** Transcription-related proteins with significantly different levels of association with immunoprecipitated RpoA

Gene name	Protein name	FC^*a*^	JN1/WT^*b*^	ToS^*c*^	*P* value
*BP0737*	Putative inner membrane sensor for iron transport	NA	**↑**	P/A	
*BP1315*	Universal stress family protein	1.7	**↑**	*t* test	0.044
*BP2216*	Putative transcriptional regulator (MarR family)	1.3	**↑**	*t* test	0.017
*fur*	Ferric uptake regulation protein	NA	**↑**	P/A	
*infA2*	Translation initiation factor IF-1 2	2.2	**↑**	*t* test	0.002
*BP2227*	Putative anti-sigma factor	NA	**↓**	P/A	
*BP3138*	Putative two-component system response regulator	1.4	**↓**	*t* test	0.046
*brpL*	Putative RNA polymerase sigma factor	NA	**↓**	P/A	
*rpoB*	DNA-directed RNA polymerase subunit beta	NA	**↓**	P/A	
*bvgA*	Transcriptional regulator BvgA	2.4	**↓**^*d*^	*t* test^*d*^	0.166

a FC, fold change difference; NA, not applicable, as the protein was not detected in the WT sample.

b JN1/WT, trend observed in JN1 mutant compared to that in the wild-type strain. **↑**, more protein was immunoprecipitated in JN1 strain; **↓**, less protein was immunoprecipitated in JN1 strain.

c ToS, type of significance analysis; P/A, significance determined based on presence/absence criterion.

d Not statistically significant.

10.1128/mSystems.00612-20.8 TABLE S7 Proteomic analysis of eluates from immunoprecipitation assay with α-RpoA antibody. Download Table S7, XLSX file, 0.2 MB. Copyright © 2020 Novák et al. 2020 Novák et al. This content is distributed under the terms of the Creative Commons Attribution 4.0 International license.

### The JN1 mutant is prone to killing in human serum and to autoaggregation but persists inside human macrophage cells and in infected mouse lungs.

**FIG 3:**
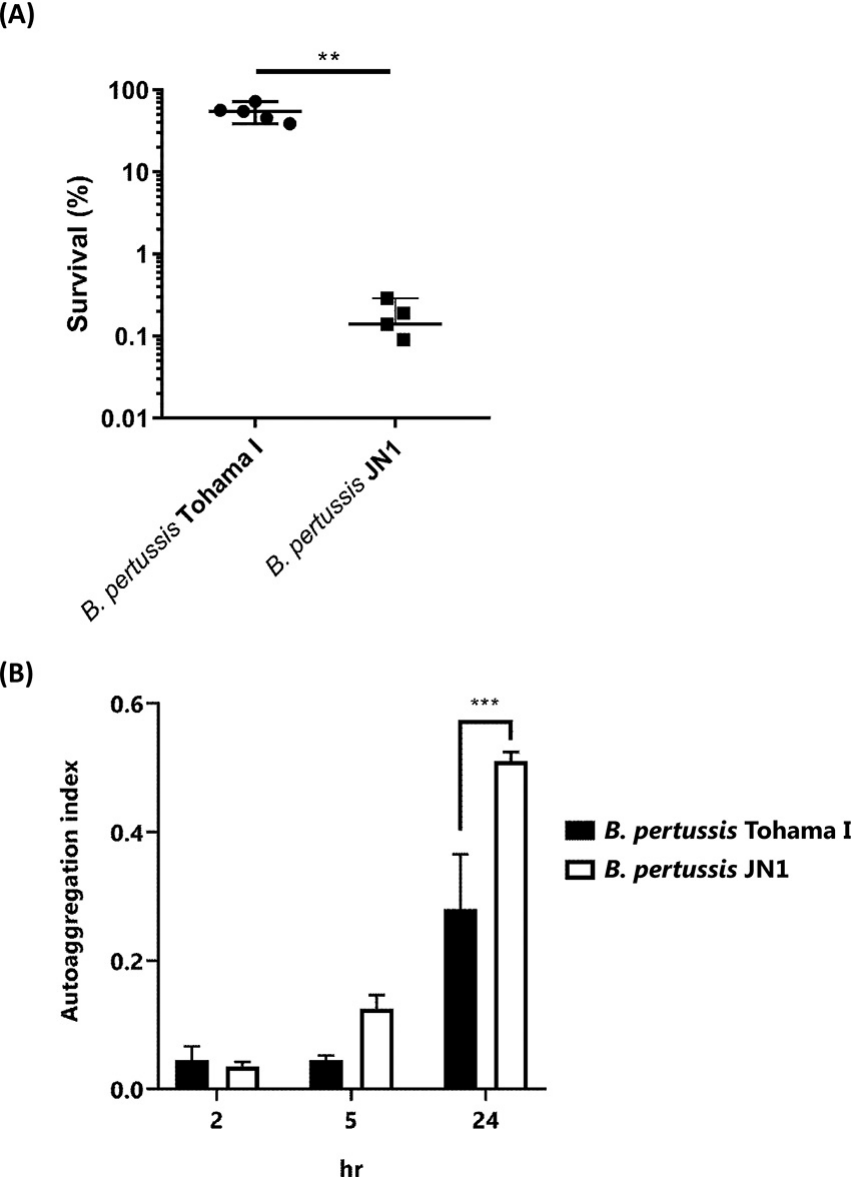
Comparison of serum resistance and autoaggregation capacities between wild-type and JN1 strains of B. pertussis. (A) Serum-killing assay with wild-type and JN1 strains of B. pertussis; 10^6^ CFU/ml were incubated in 2% normal human serum for 1 h at 37°C, and serial dilutions of the suspension were plated for CFU counting. The CFU count of bacterial suspensions incubated with heat-inactivated serum was set as 100%. (B) The autoaggregation index was determined as described in Materials and Methods. The experiments were performed 3 times in triplicates (*n* = 9) and means ± standard deviations (SD) are given. **, *P* ≤ 0.01; ***, *P* ≤ 0.001.

To corroborate the results of the omics analysis, we next assessed some of the proteome-predicted phenotypes of the JN1 mutant. Among its most downregulated virulence factors were the BrkA and Vag8 autotransporter proteins that account for resistance of B. pertussis to complement-mediated killing in human serum (53). Indeed, the JN1 mutant was approximately 10-fold more sensitive to killing in human serum than wild-type bacteria (Fig. 3A).

Conversely, the JN1 mutant exhibited an enhanced production of the Bvg-intermediate phase protein BipA (cf. Table 2) that is involved in *Bordetella* biofilm formation (35, 54) and exhibited a higher autoaggregation index than the wild-type Tohama I strain (Fig. 3B).

**FIG 4:**
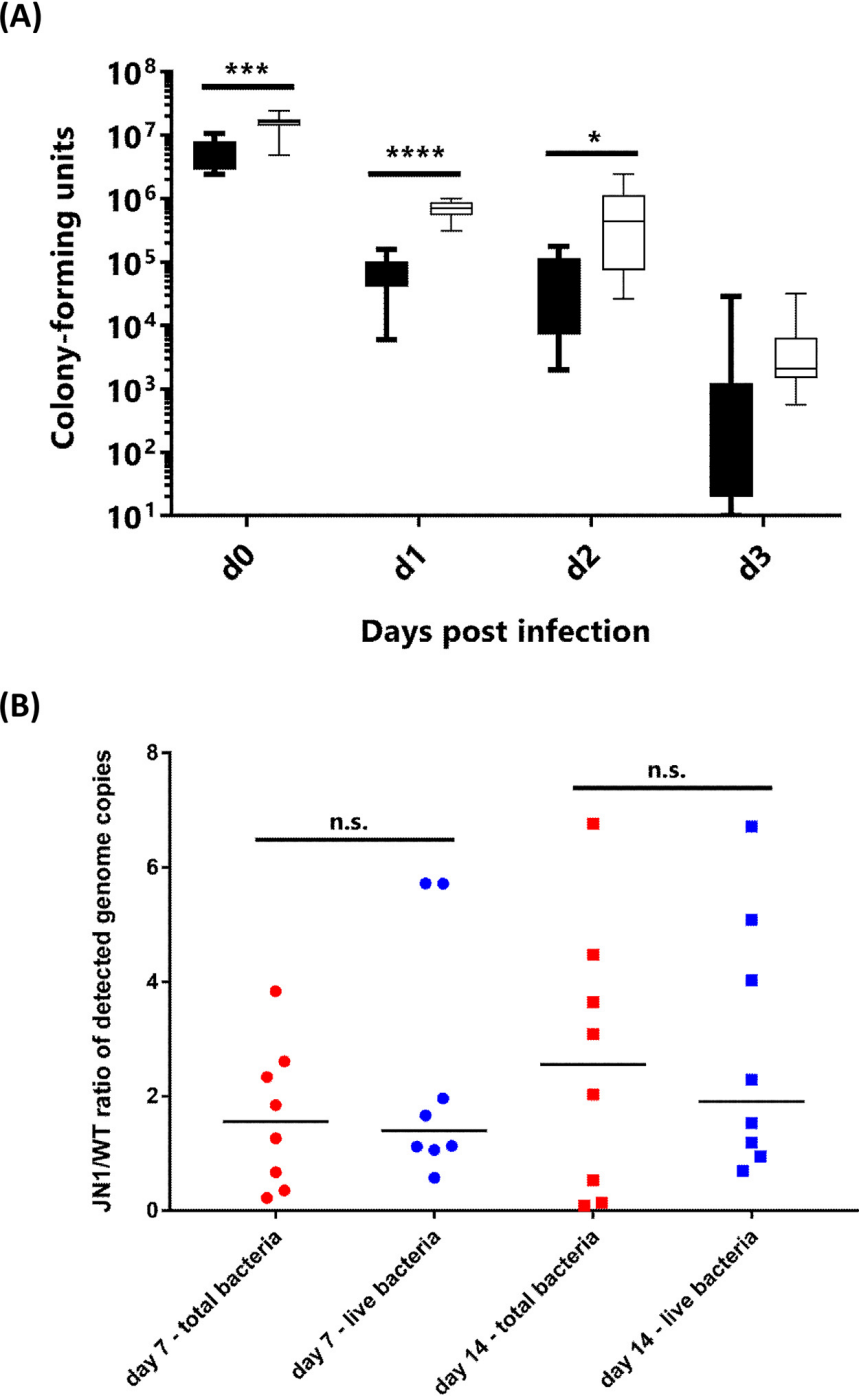
Intracellular survival of wild-type B. pertussis and of the JN1 mutant inside primary human macrophages (hPBDMs). (A) CFU counts of bacteria surviving intracellularly in infected hPBDMs for up to 3 days, as determined by plating of equal amounts of macrophage lysates on BGA. Black bars represent wild-type B. pertussis, white bars represent B. pertussis JN1. (B) Ratios of JN1/WT genome equivalents of intracellular bacteria per equal number of infected macrophage cells after 7 and 14 days of infection were determined by the propidium monoazide-based qPCR assay. Red symbols, total detected genomes; blue symbols, genomes of live bacteria capable of excluding phorbol myristate acetate (PMA). ns, *P* > 0.05; *, *P* ≤ 0.05; ***, *P* ≤ 0.001; ****, *P* ≤ 0.0001.

Intriguingly, nonopsonized B. pertussis bacteria were recently found to survive to some extent the internalization into human macrophages and to adapt to the intracellular niche by downregulating virulence factor production (39, 55, 56). As shown in Fig. 4A, despite the reduced production of adhesins, the JN1 bacteria were internalized at higher levels than the wild-type bacteria by primary human peripheral blood monocyte-derived macrophages (PBDMs), becoming protected from killing by the antibiotic polymyxin B (100 μg/ml) added into culture medium after 2 h of coincubation of bacteria with PBDMs (day 0 [d0]). Moreover, approximately one order of magnitude higher CFU numbers of the JN1 mutant than of the wild-type bacteria (Fig. 4A) were recovered from repeatedly washed macrophages that were cultured for 24 or 48 h after infection in medium containing 10 μg/ml of polymyxin B that kills any noninternalized extracellularly attached bacteria. Intriguingly, at time points beyond 72 h from infection, no bacteria able to form colonies on BGA plates were recovered from the infected macrophages. However, our parallel experiments with mScarlet-expressing fluorescent B. pertussis revealed that nonopsonized B. pertussis can persist in primary human macrophages grown in medium containing 5% human plasma for many weeks (J. Novak, unpublished data). This suggested that beyond day 3 from infection, the surviving intracellular bacteria adopted a dormant and viable but nonculturable (VBNC) state inside the macrophage cells (57). Therefore, we used a “live/dead” propidium monoazide-based qPCR assay for differential enumeration of the intracellularly surviving or killed bacteria (58). As shown in Fig. 4B, on days 7 and 14 after infection, comparable numbers of total genome equivalents of the JN1 and wild-type (WT) bacteria (live plus dead) were detected in the lysates of infected macrophages. For the fraction of viable bacteria, the JN1/WT ratio was slightly >1 (∼1.1). Hence, despite being defective in virulence factor production, the JN1 mutant persisted inside primary human macrophage cells over the prolonged period of 2 weeks as well as the wild-type bacteria.

**FIG 5:**
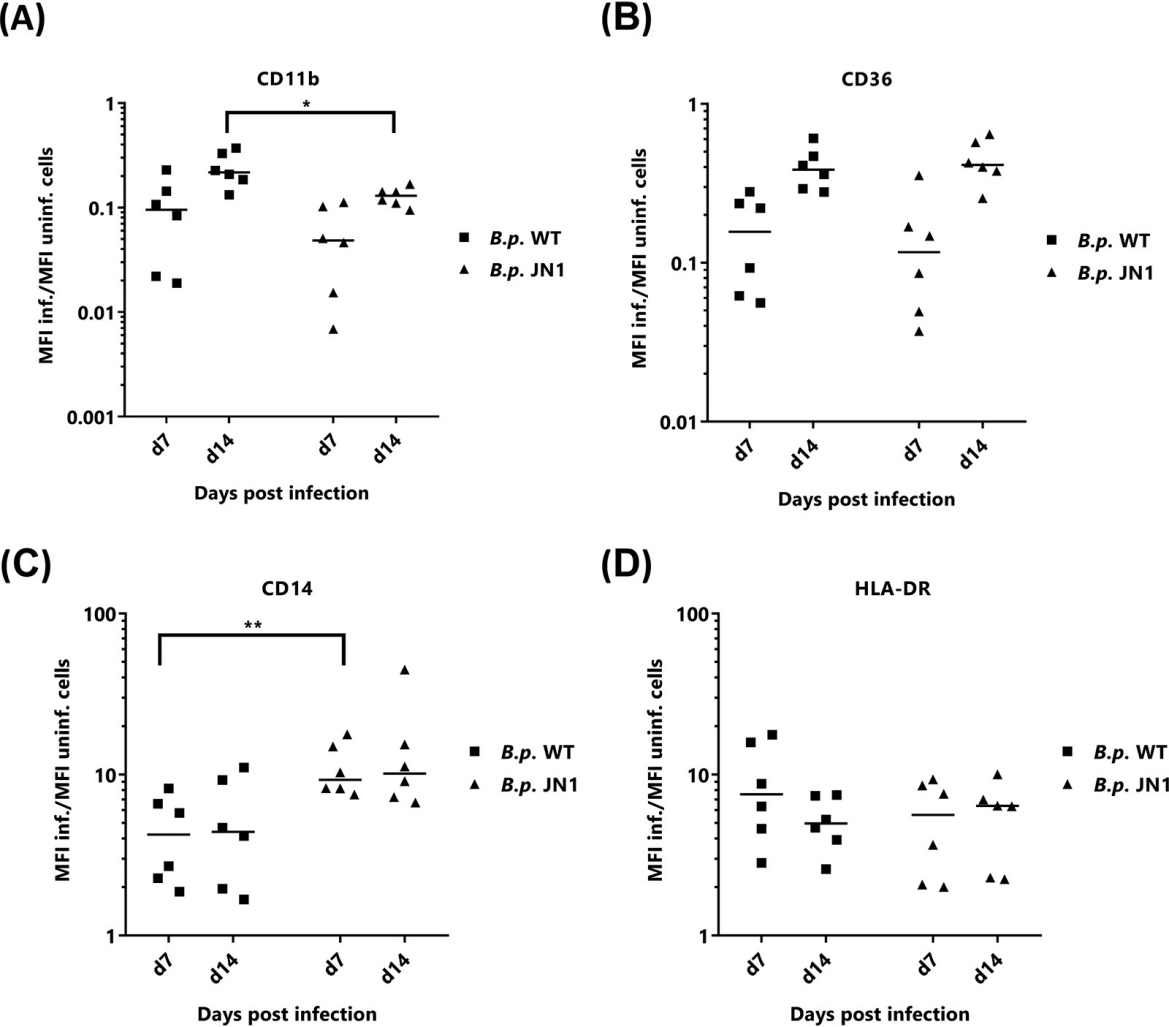
Persisting intracellular infection with wild-type or JN1 mutant B. pertussis bacteria alters expression of phenotypic markers of macrophage cells. On days 7 and 14 after infection (MOI, 50:1) of primary human peripheral blood-derived macrophages with the wild-type or JN1 mutant B. pertussis bacteria, the expression of surface markers on intracellularly infected and noninfected cells was compared by flow cytometry analysis. Comparison of mean fluorescence intensities (MFIs) of CD11b (A), CD14 (B), CD36 (C), and HLA-DR (D) surface markers are shown as ratios of MFI of infected to MFI of uninfected cells. The relative MFI value found for uninfected cells was set as 1. Differences between samples on days 7 and 14 postinfection were statistically tested by *t* test. *, *P* ≤ 0.05; **, *P* ≤ 0.01.

Since we have recently observed that long-term intracellular persistence of B. pertussis bacteria modulates the phenotype of the infected human macrophages (J. Novak, unpublished data), we compared the phenotypic changes elicited in PBDM cells by internalized wild-type and JN1 mutant bacteria (Fig. 5). As shown in Fig. 5A and B and compared to that in noninfected cells, after 7 days of intracellular persistence, both wild-type and JN1 bacteria provoked a strong and comparable (∼10-fold) downregulation of surface expression of the CD11b subunit of the complement receptor 3 (Fig. 5A) and of the CD36 class B scavenger receptor (Fig. 5B). In turn, infection by both strains provoked upregulation of cell surface expression of the lipopolysaccharide (LPS) receptor CD14 (Fig. 5C) and of the HLA-DR molecule involved in antigen presentation to T cells (Fig. 5D). The JN1 mutant then triggered exposure of significantly higher levels of CD14 than the wild-type bacteria. Moreover, the level of CD14 remained high over the 2 weeks of intracellular infection. Hence, despite a strongly reduced capacity to produce the known virulence factors in standard laboratory culture media, the JN1 mutant survived inside primary macrophage cells at least as well as the wild-type B. pertussis bacteria, and it triggered similar phenotypic changes of infected macrophage cells as the wild-type bacteria.

**FIG 6:**
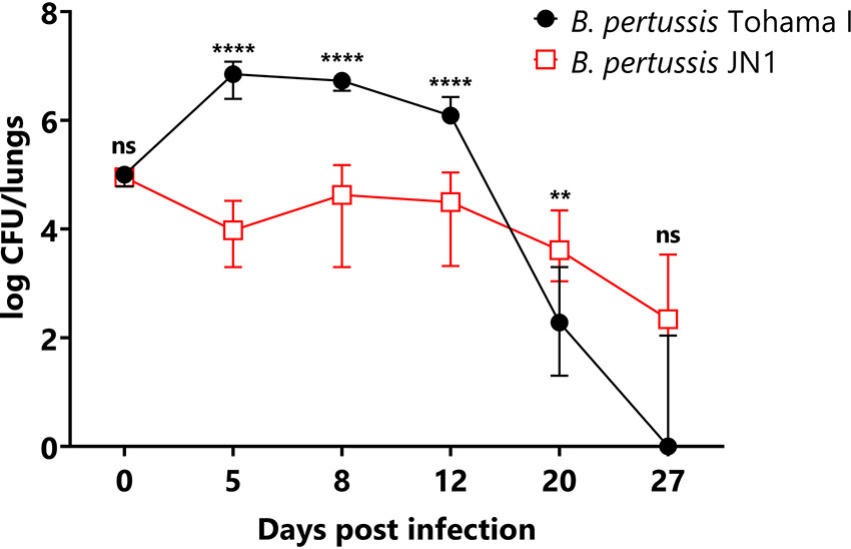
Comparison of lung colonization of infected mice between wild-type and JN1 mutant B. pertussis. Mice were inoculated intranasally with 50 μl of suspensions containing 1 × 10^5^ CFU of the B. pertussis strains. At the indicated time points, the challenged mice were sacrificed, and lung homogenates were plated on Bordet-Gengou agar for CFU counting after 5 days. The experiment was repeated twice with groups of 3 mice per time point, yielding very similar results. Therefore, the values from two experiments were pooled, and the medians (*n* = 6 for day postinfection [dpi] 0 to 27) with 95% confidence intervals (CIs) are shown. ns, not significant; *, *P* ≤ 0.05; ****, *P* ≤ 0.0001.

This prompted us to test the capacity of the JN1 mutant to colonize mouse lungs. Five-week-old female BALB/cByJ mice were intranasally challenged with 1 × 10^5^ CFU (50 μl) of the wild-type or JN1 bacteria, and lung colonization was followed over 27 days. As shown in Fig. 6, whereas the wild-type B. pertussis bacteria proliferated by 2 orders of magnitude within a week from infection before being progressively cleared from the lungs, the JN1 bacteria did not proliferate over the first week of infection but persisted longer and at higher numbers than the wild-type strain. In two independent challenge experiments yielding highly reproducible results, the persisting JN1 bacteria were still isolated from mouse lungs at a low but reliably detectable level even 27 days after infection, when the wild-type bacteria have in the majority of mice been cleared (Fig. 6).

## DISCUSSION

We report that a spontaneous G-to-T transversion in the 5′ UTR of the first gene (*rplN*) of a ribosomal operon exerted a pleiotropic effect on the global expression pattern of the B. pertussis genome. Moreover, this single G-to-T base transversion located 18 bases upstream of the ATG start codon of *rplN* provoked a generalized defect of virulence factor production, most likely through the downregulation of the levels of the BvgA∼P transcriptional activator of *Bordetella* virulence gene expression.

RNA-seq analysis revealed that the point mutation enhanced, by an as-yet-undefined mechanism, the steady-state abundance of transcripts of the *rplN-rpsD* operon and of the *rpoA* gene located immediately downstream (cf. Fig. 1). Recent analysis of the primary transcriptome of B. pertussis did not reveal any *rpoA* promoter (59). This raises the hypothesis that the *rpoA* open reading frame (ORF), starting 214 nucleotides downstream of the stop codon of the *rpsD* ORF, is cotranscribed at least to some extent with the strongly expressed ribosomal operon. Alternatively, upregulation of *rpoA* transcription from a putative own promoter or enhanced *rpoA* transcript stability may have accounted for the observed RpoA overproduction in the JN1 mutant. Since it is not obvious by which mechanism such effects could be caused by a G-to-T transversion located some 7.4 kb upstream of *rpoA* in the 5′ UTR of the *rplN* gene, the mechanism by which *rpoA* expression is increased in the JN1 mutant deserves further experimental exploration.

The observed overproduction of RpoA offers a plausible explanation of the mechanism by which the G-to-T transversion in a noncoding region of a housekeeping operon would exert such a pleiotropic effect on gene expression and virulence factor production in B. pertussis. Indeed, Carbonetti and coworkers previously observed that overproduction of RpoA due to mutations in the 5′-UTR of the *rpoA* gene resulted in reduced transcription of BvgA-activated virulence genes and could be reversed by inducible overexpression of the *bvgAS* genes in mutants overproducing RpoA (60, 61). Moreover, previous suppressor analyses revealed that the RpoA and BvgA proteins interact through their C-terminal moieties within transcriptional initiation complexes (41, 60, 62). Hence, an initial partial sequestration of the BvgA∼P protein into nonproductive complexes by the overproduced RpoA would reduce, first of all, the transcription of the *bvgAS* locus itself, as this is positively autoregulated by the transcriptional activator BvgA∼P produced by phosphorylation of BvgA by BvgS (23). Hence, the more BvgA∼P is withdrawn from the transcription initiation complexes of RNA polymerase by the excess of RpoA subunit, the smaller are the amounts of the BvgA and BvgS proteins produced *de novo* in the JN1 cells. Hence, the concentration of BvgA∼P in cells will drop further and become limiting for activation of transcription of the BvgA∼P-dependent virulence genes (41, 42, 60–62). Indeed, an approximately 3-fold lower concentration of BvgA∼P was detected in JN1 cells by Western blots of Phos-tag gel-resolved BvgA isoforms (cf. Fig. 2). This result lends support to the operation of a downregulating loop of decreasing activation of *bvgAS* transcription (cf. Table 2).

It remains to be determined by which mechanism the G-T base transversion in the 5′-UTR of the *rplN* gene increases the steady-state abundance of transcripts of the *rplN-rpsD* operon. The G-T transversion occurred at a site where no sRNA molecule sequence was predicted, and the performed RNA-seq analysis did not reveal the existence of any sRNA molecule that would have been affected by the analyzed mutation.

While it is plausible to conclude that the virulence genes were downregulated in the JN1 mutant through downregulation of BvgA∼P production, the G-T transversion in the 5′ UTR of the *rplN* gene exerted a much broader effect on the global expression profile of the B. pertussis genome. It is plausible to assume that downregulation of the BvgAS system in the JN1 mutant allowed production of *vrg* proteins that are involved in transcriptional activation of other B. pertussis regulons. Nevertheless, the extent of BvgA downregulation in the JN1 mutant was not sufficient for a full upregulation of all of the >300 previously identified *vrg* genes transcribed when B. pertussis is in the Bvg^−^ phase (23, 49, 63). Expression of only 144 *vrg* genes was significantly upregulated (FC ≥ 1.5, *P* < 0.05) in JN1, and the already very low residual transcription level of some 37 *vrg* genes was even lower (FC ≥ 1.5, *P* < 0.05) in JN1 than in the wild-type strain (see Table S6 in the supplemental material). For example, the *vrg-6* gene was not upregulated in JN1 (Table 3), most likely because of the insufficient downregulation of the BvgR phosphodiesterase that hydrolyzes c-di-GMP and thereby prevents activation of *vrg-6* transcription by the c-di-GMP-activated RisA (34). Furthermore, the mixed pattern of transcription of *vrg* genes in JN1 did not confer a true Bvg^−^ phenotype. It is plausible to assume that many transcription factors other than BvgA∼P also intervened in the regulation of expression of *vrg* genes.

Indeed, among the transcriptional regulators produced at increased amounts in the JN1 mutant were the proteins encoded by the *BP2216*, *BP2913*, *BP1315*, and *fur* genes. *BP2216* encodes a transcriptional regulator of the MarR family (23, 39), and *BP2913* encodes a predicted anti-sigma factor, similar to the RslA and RskA anti-sigma factors of Mycobacterium tuberculosis (64, 65). The *BP1315* gene-encoded protein belongs to the family of universal stress proteins. The ferric uptake regulation (Fur) protein encoded by the *fur* gene primarily serves under iron-replete conditions as a transcriptional repressor of dozens of iron-regulated genes and orchestrates expression of iron acquisition and homeostasis systems of bacteria. In accordance with such a major regulatory role of Fur, and because of its capacity to bind the RNA polymerase (66), it is plausible to assume that its enhanced production contributed to the generalized deregulation of gene expression in the JN1 mutant.

Another layer of gene expression deregulation may have occurred on the posttranscriptional level. For example, overproduction of RpoA was associated with a significant downregulation of a highly conserved 86-kDa protein possessing both RNA and DNA binding domains and initially named as Tex for “toxin expression protein” (Table 2). Tex was previously shown to bind the 5′ regions of target mRNAs and was previously found to positively regulate the production of a set of toxins in C. perfringens (47, 67). Moreover, Tex also appears to be involved in the regulation of virulence of Burkholderia pseudomallei, and it was initially discovered by Fuchs and coworkers as a dominant antisuppressor that ablated the effect of an as-yet-uncharacterized suppressor mutation that restored PT and CyaA production in B. pertussis mutants overproducing RpoA (47, 48). Tex, hence, appears to be somehow involved in regulation of virulence factor production in B. pertussis, and its downregulation may have affected the composition of the JN1 proteome.

Somewhat surprisingly, we observed that despite a generalized defect in virulence factor production, the JN1 mutant infected primary human macrophages at least as efficiently as the wild-type bacteria and persisted at comparable levels within macrophages for at least 2 weeks. This goes well with the recent observation that adaptation of B. pertussis to the intracellular niche inside human macrophages is accompanied by a downregulation of most of the known virulence factors (39). Indeed, the persistent intracellular infection by the JN1 bacteria with impaired virulence factor production triggered similar alterations of expression of macrophage markers as infection by the wild-type bacteria capable of producing the virulence factors, with the exception of the significantly higher upregulation of the CD14 marker by JN1 infection.

Moreover, despite the impairment in virulence factor production and despite increased sensitivity to complement-mediated killing in human serum, the JN1 mutant was still able to persistently infect mouse lungs at reduced but well-detectable levels (cf. Fig. 6). It will be of interest to determine if the upregulated expression of the autotransporter protein BatB contributed to the survival of the JN1 mutant in mouse lungs, since a homolog of BatB was found to protect B. bronchiseptica from inflammatory clearance from the mouse respiratory tract (68). The persistence of the JN1 mutant further indicates that downregulation of production of the known virulence factors may allow B. pertussis low-level colonization of the airway mucosa without harnessing the inflammatory and immune responses of the host. This would go well with the intriguing observation that persistent infection of the airways of primates was accompanied by accumulation of Bvg^−^ phase-locked B. pertussis mutants (37, 38). Moreover, B. pertussis antigen was detected inside columnar epithelial cells of the bronchiole and trachea of an infant 8 weeks after a diagnosis of pertussis (69). The sum of these observations raises the possibility that modulation of BvgAS activity or Bvg phase variation may play a role also in the lifestyle of B. pertussis, possibly during an intracellular stage of airway infection.

## MATERIALS AND METHODS

### B. pertussis strains and growth conditions.

B. pertussis JN1 was derived from the wild-type Tohama I strain obtained from the Institute Pasteur collection (CIP 81.32). The JN1 mutant was constructed by introduction of the G→T base transversion at position 3,838,664 of the B. pertussis Tohama I chromosome through markerless allelic exchange, using the pSS4245 vector (gift from Scott Stibitz). Briefly, an SpeI-EcoRI site-flanked upstream DNA fragment, carrying the G→T base transversion, was amplified using the primer pair 5′-ACTAGTGTGGCCCTGGTCGTGCTG-3′ (forward) and 5′-GAA-TTCCGGCAGCAAGCACACCAGCGTGATGTAAATCGCAA-3′ (reverse). The EcoRI-BamHI site-flanked downstream fragment was amplified using the primers 3′-GAATTCCTGGTGATGCGTTG-3′ (forward) and 5′-GGATCCCACCACGCCGTGACGTTTG-3′. The two PCR products were digested by the corresponding restriction endonucleases and simultaneously ligated into the pSS4245 plasmid linearized by SpeI and BamHI. The construct carrying the mutation was verified by sequencing, and the cloned fragment was next recombined into the bacterial chromosome by double crossing over, according to a previously described procedure (70). The introduction of the mutation into a bacterial chromosome and absence of undesired mutations were then verified by PCR resequencing. Bacteria were grown on Bordet-Gengou (BG) agar plates supplemented with 15% defibrinated sheep blood (LabMedia Servis, Czech Republic) at 37°C and 5% CO_2_ for 5 days to visualize hemolysis. Liquid cultures were obtained by growing bacteria in modified Stainer-Scholte medium supplemented with 3 g/liter of Difco Casamino Acids (Thermo) and 1 g/liter of heptakis(2,6-di-*O*-dimethyl)-β-cyclodextrin for 18 h at 37°C.

### Genome sequencing, annotation, and SNP detection.

The genome of the JN1 strain was sequenced at 171-fold coverage using Illumina MiSeq system, and the adaptor sequences and low-quality bases were removed from the reads using the Trimmomatic tool (71). The reads were mapped to B. pertussis Tohama I genome using Burrows-Wheeler Aligner (72). Uniquely mapped reads were selected using SAMtools (73). Single-nucleotide polymorphisms (SNPs) and insertions/deletions in the studied genome compared to that of B. pertussis Tohama I were detected with Pilon software (74). The data for this study have been deposited in the European Nucleotide Archive (ENA) at EMBL-EBI under accession number PRJEB38438.

### Quantitative PCR analysis of *rpoA* transcription.

Reverse transcription-quantitative PCR (RT-qPCR) analysis of the *rpoA* transcript levels was performed as described earlier (75). Samples of total RNA (biological triplicates of RNA isolated from WT and JN1 strains used for RNA-seq analysis) were treated using the TURBO DNA-free kit (Thermo Fisher Scientific). Aliquots of total RNA (1 μg) were reverse transcribed in duplicates into cDNA in a 25-μl reaction mixture using a reverse transcription system (Promega). All primers (see Table S8 in the supplemental material) were designed to anneal at 60°C and were analyzed for secondary structures and for cross-dimers in primer pairs. RT-qPCR analysis of each cDNA sample was performed in technical triplicates on a Bio-Rad CFX96 instrument using SYBR green JumpStart *Taq* ReadyMix (Sigma-Aldrich), 200 nmol of each primer, and 40 ng of reverse transcribed RNA in a 20-μl reaction mixture. The *rpoB* gene was used as the reference gene, and relative gene expression was quantified using amplification efficiency values (76).

10.1128/mSystems.00612-20.9 TABLE S8 Lists of RT-qPCR primers used for *rpoA* quantification and antibodies used for fluorescence-activated cell sorting analysis. Download Table S8, XLSX file, 0.01 MB. Copyright © 2020 Novák et al. 2020 Novák et al. This content is distributed under the terms of the Creative Commons Attribution 4.0 International license.

### Analysis of RpoA protein levels by Western blotting.

Wild-type and JN1 B. pertussis bacteria were grown in 3 ml of SSM to an optical density at 600 nm (OD_600_) of approximately 1.1. Cells from 1 ml of culture were harvested by centrifugation at 16,000 × *g* for 5 min at room temperature, and bacterial pellets were resuspended in 400 μl of TUS buffer (50 mM Tris-HCl [pH 8.0], 8 M urea, 2% [wt/vol] SDS). Samples were heated for 5 min at 95°C, and equal amounts of protein of the JN1 and WT samples were resolved by 10% SDS-PAGE, transferred onto the nitrocellulose membrane, and probed with a 1:3,000 diluted rabbit anti-RpoA serum kindly provided by Linda Doubravova (52). Blots were incubated with a peroxidase-labeled secondary anti-rabbit antibody at a 1:5,000 dilution, and the RpoA protein was detected using enhanced chemiluminescence (ECL) with a SuperSignal West Femto maximum sensitivity substrate (Thermo Scientific). The chemiluminescent signals were quantified using the Fiji processing package of ImageJ (77).

### Infections of primary human PBDMs.

Human peripheral blood mononuclear cells from anonymous healthy donors were isolated from buffy coats purchased at Thomayer Hospital (Prague, Czech Republic). The cells were purified using a modified double-gradient centrifugation method, as described by Menck et al. (78), with the modification that fetal calf serum was replaced by heat-inactivated (56°C, 30 min) 5% human AB serum (HS) obtained from pooled plasma from 5 male donors (Thomayer Hospital, Prague). For long-term culture experiments, the medium with HS was systematically sterilized by filtration with 0.22-μm filters before use. Differentiation was performed in Petri dishes for bacterial cultures for six to 7 days in RPMI medium (Sigma) with 5% HS, supplemented with 50 ng/ml recombinant human macrophage colony-stimulating factor (rh M-CSF; ImmunoTools) with antibiotics; half of the medium was changed after 3 to 4 days. The freshly added medium contained 50 ng/ml rh M-CSF but no antibiotics. One day before infection, the cells were washed twice with phosphate-buffered saline (PBS), detached by incubation with 0.02% EDTA solution for 15 min at 37°C, collected by centrifugation at 1,300 rpm for 3 to 5 min, resuspended in Dulbecco’s modified Eagle medium (DMEM) without antibiotics (Sigma) and containing 5% HS, counted, and seeded into 12-well plates at ∼0.4 × 10^6^ cells per well.

Bacterial inocula were grown as liquid cultures to an OD_600_ of 1.0 to 1.3 (∼2 × 10^9^ CFU/ml), diluted in SS medium, and used to infect macrophage cells at a multiplicity of infection (MOI) of 50:1. Culture plates were centrifuged at 640 × *g* for 5 min to facilitate bacterial attachment to macrophage cells, followed by coincubation for 2 h at 37°C in a humidified 5% CO_2_ atmosphere. Unattached bacteria were washed away with prewarmed PBS, and cells were replaced in medium containing 100 μg/ml of polymyxin B for 1 h to kill noninternalized bacteria. The cells were next washed with PBS and either lysed for CFU plating (d0) or cultured for determined times in medium containing 5% HS and 10 μg/ml of polymyxin B to block extracellular bacterial growth. Equal numbers of cells were lysed by resuspension in 900 μl of sterile water, followed by addition of 100 μl of 10× PBS. The lysates were serially diluted in PBS and plated on BGA for determination of B. pertussis CFU after 5 days of growth.

### Live/dead quantitative real-time PCR.

A modified version of the protocol described by Ramkissoon et al. (58) was used. Macrophage cells grown in 12-well plates were washed with PBS, detached in 1 ml of 0.02% EDTA for 15 min at 37°C, resuspended by scraping, and collected by centrifugation at 2,000 × *g* for 10 min. Cell pellets were resuspended to equal cell density in PBS, and 500 μl of cell suspensions were split into two 200-μl aliquots. One microliter of 20 mM PMAxx (Biotium, USA) was added to one aliquot, and both aliquots of a sample were incubated in the dark at room temperature on a rocking platform for 10 min. All cell samples were next irradiated for 5 min in the PMA-Lite light-emitting diode (LED) photolysis device (Biotium, USA) and eventually frozen. Genomic DNA from macrophages was isolated using the QIAamp DNA minikit (Qiagen) according to the manufacturer’s instructions. For qPCR analysis, the TaqMan gene expression master mix (Applied Biosystems) was used. The 20-μl qPCR mixtures for quantification of the IS*481* target sequence contained 10 μl of TaqMan master mix, 2 μl of each 900 nM primer, 2 μl of the 150 nM probe, and 4 μl of template, using the IS481_fwd ATCAAGCACCGCTTTACCC and IS481_rev TTGGGAGTTCTGGTAGGTGTG primers and the IS*481* probe 6-carboxyfluorescein (FAM)-AATGGCAAGGCCGAACGCTTCA-black hole quencher 1 (BHQ1). Bacterial genomic DNA was used as a positive control, and the total number of detected B. pertussis genome copies was calculated as described by Ramkissoon et al. (58). Bacterial survival at the time points 7 and 14 days of infection was calculated as a ratio between the numbers of genomes detected in macrophages after the infection with JN1 mutant strain compared to that with wild-type B. pertussis.

### Flow cytometry analysis.

Cells were stained with specific antibodies or isotype controls (Table S8) in 96-well plates on ice and analyzed by flow cytometry using a BD LSR II instrument with high-throughput sampler and evaluated using FlowJo V10 software.

### Analysis of BvgA and BvgA∼P protein levels.

Wild-type and JN1 B. pertussis bacteria were cultured in SSM to an OD_600_ of ∼1, cells from 1 ml were collected by centrifugation, and whole bacterial cell lysates were prepared and analyzed as described by Chen at al. (50). Based on the individual OD_600_ values of each culture, the sample volumes were adjusted to load equal amounts of total bacterial protein onto 12.5% SuperSep Phos-tag gels (Wako). The resolved BvgA and BvgA∼P isoforms were detected by Western blotting using a rabbit polyclonal anti-BvgA antibody (51) with chemiluminescence detection. The total amount of BvgA protein was calculated by summing up the signal of both bands (phosphorylated and unphosphorylated) from densitometry analysis. To determine the ratio of the phosphorylated and unphosphorylated forms of BvgA, the chemiluminescent signal from the upper band was divided by the chemiluminescent signal from the lower band in the given lane.

### Proteomic analysis.

Bordetella pertussis Tohama I and JN1 strains grown on BGA plates for 3 days at 37°C with 5% CO_2_ were used to inoculate 5 ml of liquid cultures in SS medium to a final OD_600_ of 0.2. Bacterial cultures were grown overnight at 37°C and eventually inoculated 1:10 into 50 ml of SSM. After reaching the late-exponential phase (OD of ∼1), the cultures were centrifuged at 20,000 × *g* for 20 min at 4°C, and for the analysis of the secretome, 35 ml of the upper portion of the supernatant was carefully withdrawn from each tube. The remaining bacteria were removed by passage of the supernatants through a 0.22-μm filter, and trichloroacetic acid was added to a final concentration of 10%. The proteins were precipitated overnight at 4°C and collected at 20,000 × *g* for 20 min at 4°C, and the precipitate was washed with ice-cold acetone. For proteomic analysis of whole bacterial cells, bacterial pellets were washed in PBS. Both washed cells and the precipitates of the supernatants were resuspended by 8 M urea in 50 mM ammonium bicarbonate buffer, pH 8.3 (UA buffer). Three biological replicates of each sample were subjected to liquid chromatography tandem mass spectrometry (LC-MS/MS) proteomic analysis.

For analysis of composition of RpoA complexes, ∼3 × 10^10^ B. pertussis cells were lysed in 0.5 ml of buffer, and immunoprecipitation was performed with a rabbit anti-RpoA serum kindly provided by Linda Doubravova (52), using a Pierce co-immunoprecipitation (Co-IP) kit (catalog number 26149; Thermo Scientific), according to the manufacturer’s instructions. Pierce control agarose resin was used as a negative control. Three biological replicates of each sample were analyzed by LC-MS/MS.

Briefly, 70 μg of total protein of whole bacterial cells, from culture supernatant precipitate, or of immunoprecipitate was applied to a 30-kDa-cutoff membrane filter and processed using a modified protocol for filter-aided sample preparation (FASP) (79). Briefly, proteins were washed twice by UA buffer and then by 50 mM ammonium bicarbonate buffer, pH 8.3 (AB buffer). Reduction of disulfide bonds was carried out by 100 mM dithiothreitol (DTT) in AB buffer (reduction buffer) on a shaker for 30 min at 60°C at 200 rpm followed by alkylation of sulfhydryl groups by the addition of chloroacetamide (CAA) to final 50 mM concentration for 30 min at room temperature in the dark. Solutions were discarded, and mass spectrometry-grade trypsin in AB buffer was added onto the filter at a protein/enzyme ratio of 35:1. The overnight incubation on a shaker at 37°C and 200 rpm then followed. Afterwards, peptides were eluted by three consecutive centrifugations with AB buffer at 14,000 × *g* for 20 min and acidified by 2% trifluoroacetic acid (TFA) to reach a final concentration of 0.2% TFA. Peptides were then desalted using C_18_ extraction disks (Empore, USA) and filled ZipTip tips and vacuum dried. Prior to LC-MS/MS analysis, samples were resuspended in 2% acetonitrile (ACN) in 0.1% TFA.

A nano reversed-phase column (EASY-Spray column, 50 cm, 75-μm inside diameter [i.d.], PepMap C_18_, 2-μm particles, 100 Å pore size) was used for LC-MS analysis. Mobile phase buffer A was composed of water and 0.1% formic acid. Mobile phase B was composed of acetonitrile and 0.1% formic acid. Samples were loaded onto the trap column (Acclaim PepMap300, C_18_, 5 μm, 300 Å wide pore, 300 μm by 5 mm) at a flow rate of 15 μl/min. Loading buffer was composed of water, 2% acetonitrile, and 0.1% trifluoroacetic acid. Peptides were eluted with gradient of B from 4% to 35% over 60 min at a flow rate of 300 nl/min. Eluting peptide cations were converted to gas-phase ions by electrospray ionization and analyzed on a Thermo Orbitrap Fusion (Q-OT-qIT). Survey scans of peptide precursors from 350 to 1,400 *m/z* were performed at 120,000 resolution (at 200 *m/z*) with a 5 × 10^5^ ion count target. Tandem MS was performed by isolation at 1.5 Th with the quadrupole higher-energy collisional dissociation (HCD) fragmentation with normalized collision energy of 30, and rapid scan MS analysis in the ion trap. The MS 2 ion count target was set to 104, and the max injection time was 35 ms. Only those precursors with a charge state of 2 to 6 were sampled for MS 2. The dynamic exclusion duration was set to 45 s with a 10-ppm tolerance around the selected precursor and its isotopes. Monoisotopic precursor selection was turned on. The instrument was run in top speed mode with 2-s cycles (80). The data were analyzed and quantified with label-free quantification (LFQ) algorithms in MaxQuant v1.6.3.3 (81) and the Andromeda search engine (82). Extracted ion current ratios of every detected peptide were calculated, and peptide ion signals were normalized to the dominant population of proteins that varied minimally within our experimental data sets (83). The false-discovery rate (FDR) parameter was set to 1% for both proteins and peptides. The enzyme specificity of trypsin was set as C terminal to Arg and Lys. Carbamidomethylation was set as the fixed modification, while N-terminal protein acetylation and methionine oxidation were variable modifications. Maximal number of missed cleavages was set to 2. All hits identified in searches as contaminants were filtered out. The data were searched against Bordetella pertussis reference proteome database (strain Tohama I/ATCC BAA-589/NCTC 13251).

### Statistical testing of proteomics data.

For analysis of composition of the immunoprecipitates, all proteins detected in the negative control were subtracted. Only proteins detected in at least two replicates of at least one of the strain samples were considered. Differentially regulated proteins in wild-type and JN1 mutant samples were identified by Student’s two-sample *t* test (s0 = 0.1, permutation-based FDR ≤ 0.05) applied on signal intensities. Testing was performed in Perseus ver. 1.6.5 (84). Only proteins quantified in at least two biological replicates of the wild-type or JN1 samples were tested. Proteins quantified in at least two replicates of samples from one strain but consistently absent across all three replicates of the sample from the other strain were evaluated as significantly regulated. For proteins that were significantly differentially regulated, fold change differences (FC) were calculated from the median values as FC = 2^(median_WT − median_JN1)^.

### RNA-seq analysis.

Liquid bacterial cultures (15 ml) were grown overnight as stated above. Eight-milliliter cultures at an OD of 1 to 1.5 were mixed with 2 ml of STOP solution (5% phenol in ethanol [EtOH]) and centrifuged (15 min, 8,000 rpm, 4°C), and cell pellets were frozen at −80°C until use. The pellets were thawed, lysed in 300 μl of Tris-EDTA (TE) buffer with lysozyme for 5 min at room temperature, and dissolved with 0.9 ml of the TRI reagent for 5 min at room temperature. The samples were centrifuged (10 min, 12,000 rpm, 4°C), the supernatants (1 ml) were extracted with 200 μl of chloroform, and 500 μl of the water phase was precipitated with 500 μl of isopropanol for 45 min at 4°C. Precipitates were washed twice with 900 μl of 75% EtOH and resuspended in 50 μl of RNase-free water.

rRNA was removed from 400 ng of total RNA with NEBNext rRNA depletion kit (Escherichia coli) (New England Biolabs, Ipswich, MA). Subsequently, stranded total RNA-seq libraries were prepared from rRNA-free RNA samples using the NEBNext Ultra II directional RNA library prep kit for Illumina (New England Biolabs). Small RNA-Seq libraries were prepared manually from 200 ng of total RNA using the NEBNext Multiplex small RNA library prep kit (New England Biolabs).

Obtained libraries that passed the quality control (QC) step assessed on the Agilent Bioanalyzer system were pooled in equimolar amounts. Each pool of libraries was loaded on the Illumina sequencer HiSeq 2500 and sequenced unidirectionally, generating ∼180 million reads per lane, each at 62 bases long. Raw data were deposited as project PRJEB39011 in the European Nucleotide Archive (ENA).

The adapter sequence AGATCGGAAGA and reads shorter than 10 nucleotides were removed with Cutadapt (85). The analysis was performed in R programming language using Bioconductor (86). FASTQ files were aligned using the Rsubread package version 1.34.7 (87). Reads were counted with the featureCounts software (88) that is part of the Rsubread package and Bordetella pertussis Tohama I genome assembly ASM19571v1.45. Differential expression analysis was performed with DESeq2 (89). For identification of novel and existing sRNAs, transcript boundaries, and operons, data were analyzed by Rockhopper (44, 45).

### Infection of mice.

All animal experiments were approved by the Animal Welfare Committee of the Institute of Microbiology of the ASCR, v. v. i., in Prague, Czech Republic. Handling of animals was performed according to the Guidelines for the care and use of laboratory animals, the Act of the Czech National Assembly, collection of laws numbers 246/1992. Five-week-old female BALB/cByJ (Charles River, France) were used in this study. Mice were anesthetized by intraperitoneal (i.p.) injection of ketamine (80 mg/kg of body weight) and xylazine (8 mg/kg of body weight) in saline and were inoculated intranasally with 1 × 10^5^ CFU of B. pertussis cells in 50 μl. Viable CFU were determined by plating on BG agar plates.

Infected mice were euthanized 2 h after exposure to challenge suspension (day 0 plus 2 h) and on the indicated days thereafter (days 5, 8, 12, 21, and 27). The lungs were aseptically removed and homogenized in physiological solution with tissue grinders. Serial dilutions of lung homogenates were plated on BG agar plates supplemented with 15% defibrinated sheep blood, and CFU were counted after 5 days of incubation at 37°C. Three mice per time point were used, and this experiment was repeated 2 times for data points D5 to D20 and 3 times for data point D27. The average value of the results obtained with a challenge dose of 10^5^ CFU is shown.

### Autoaggregation assay.

The autoaggregation assay was performed according to Cattelan et al. (90). Bacteria were cultured in 3 ml of SS medium with heptakis(2,6-di-*O*-methyl-β-cyclodextrin) (1 g/liter) and supplement for 24 h. Cells were harvested by centrifugation (5,000 rpm, 10 min) and resuspended in 3 ml of SS medium (with supplement, no cyclodextrin) at an OD_650_ of 1.0, followed by static incubation at room temperature. After 2, 5, and 24 h of incubation, 200 μl of the medium was taken from the top layer of the suspension, and the absorbance at 650 nm was measured. The autoaggregation index (AI) represents the fraction of aggregated cells relative to the total cell population and was calculated as follows: (ODt_0_ − OD_t_)/ODt_0_, where ODt_0_ is the initial OD measurement and OD_t_ is the OD measured at a designated time point t (2, 5, and 24 h).

### Serum resistance assay.

Precultures of liquid bacterial cultures were grown in modified SSM medium to an OD of ∼0.8 in a total volume of 20 ml, and 1 ml of cultures at an OD of 0.05 were prepared and diluted 10 times in a total volume of 1 ml of SSM. Fifty microliters of each diluted culture was then mixed with 440 μl of SSM and either 10 μl of normal human serum (Merck/Sigma-Aldrich, H4522) or 10 μl of heat-inactivated (56°C, 30 min) serum. Samples were incubated for 1 h at 37°C with shaking (300 rpm), and 50 μl was withdrawn and mixed with 450 μl of PBS containing 10 mM EDTA to inhibit the complement. Serial dilutions were plated on BG blood agar plates, and CFU were counted after 5 days of incubation.

### Data availability.

The genome sequence data of the nonhemolytic mutant have been deposited in the European Nucleotide Archive (ENA) at EMBL-EBI under accession number PRJEB38438. Data from RNA-seq analyses are deposited as the project PRJEB39011 in the European Nucleotide Archive (ENA). The mass spectrometry proteomics data have been deposited to the ProteomeXchange Consortium via the PRIDE (91) partner repository with the data set identifier PXD020023.
