# Supplementary figures and images for: A Mutation Upstream of the rplN-rpsD Ribosomal Operon Downregulates Bordetella pertussis Virulence Factor Production without Compromising Bacterial Survival within Human Macrophages
Source: mSystems. 2020 Dec 8;5(6):e00612-20. doi: 10.1128/mSystems.00612-20 (PMC7742992; doi:10.1128/mSystems.00612-20)

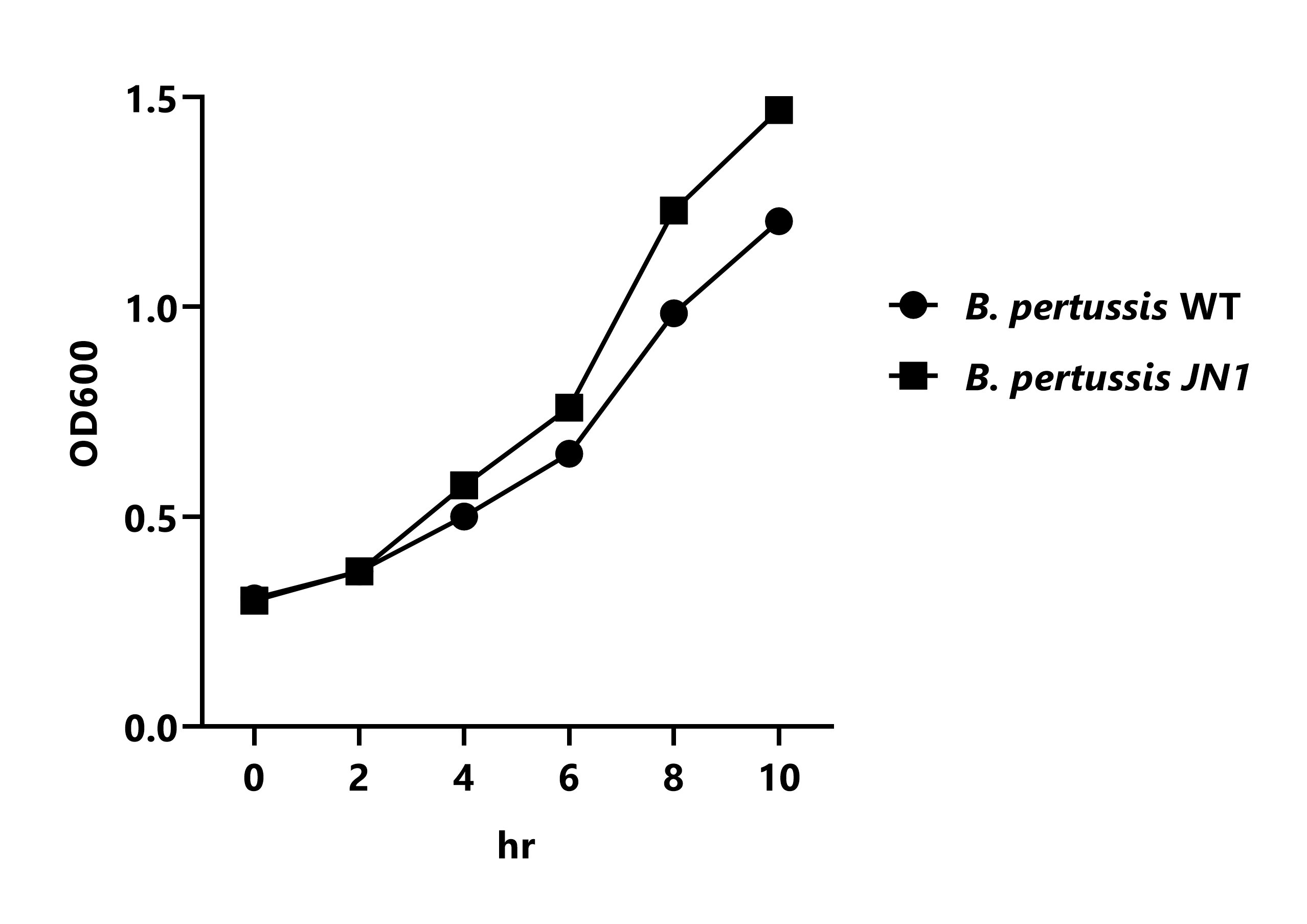

Supplement: FIG S1 [file mSystems.00612-20-sf001.tif]
